# Effect of Crack Patterns in Calcified Plaque on Lumen Area after Stenting for a Severe Calcified Coronary Artery (from the Optical Frequency Domain Imaging-Guided Percutaneous Coronary Artery Intervention for Calcified Lesion Registry)

**DOI:** 10.1155/2022/7821956

**Published:** 2022-02-27

**Authors:** Hirooki Higami, Hiroaki Matsuda, Hikaru Tateyama, Yoriyasu Suzuki, Kazuaki Kaitani

**Affiliations:** ^1^Department of Cardiovascular Medicine, Japanese Red Cross Otsu Hospital, Otsu, Shiga, Japan; ^2^Department of Cardiology, Nagoya Heart Centre, Nagoya, Aichi, Japan; ^3^Department of Clinical Engineering, Japanese Red Cross Otsu Hospital, Otsu, Shiga, Japan

## Abstract

**Background:**

Severely calcified coronary artery stenting remains a challenge due to stent thrombosis, target vessel failure, and higher mortality. Moreover, optimal vessel preparation for calcified plaque with a crack formation pattern has not been established yet. We aimed to identify the effect of crack formation in calcified plaque in the coronary artery on the lumen area after stenting.

**Materials and Methods:**

We evaluated 50 consecutive patients undergoing drug-eluting stent implantation for severely calcified lesions by using optical frequency domain imaging (OFDI) (54 lesions); we analyzed OFDI image slices every 3 mm and evaluated the segments of 242 images in those who had the arc of calcium more than 180°. Crack formation in calcified plaque was classified into three types: type 0, no cracks; type 1, no dissection between calcified plaque and vessel wall; and type 2, any dissection between calcified plaque and vessel wall.

**Results:**

Type 2 had a significantly higher area expansion ratio between preballooning and poststenting (type 0, 196% (interquartile range (IQR), 163–244); type 1, 210% (IQR, 174–244); type 2, 237% (IQR, 203–294)).

**Conclusions:**

The dissection between calcified plaque and vessel wall was a significant factor affecting lumen area expansion after stenting.

## 1. Introduction

Percutaneous coronary intervention (PCI) outcomes have improved with the development of drug-eluting stent (DES) [1–4]. However, stenting for a severely calcified coronary artery has several clinical risks, such as stent thrombosis, target vessel failure, and higher mortality [5–10]. Despite the improvement in devices and techniques, optimal vessel preparation for calcified plaque with a crack formation pattern has not been clearly established. Optical frequency domain imaging (OFDI) is an intracoronary imaging device used to evaluate the formation of calcified plaque. OFDI can clearly detect calcium distribution and crack formation of calcified plaque. The disadvantage of OFDI is that the entire vessel measurement is difficult due to signal attenuation. On the other hand, intravascular ultrasound (IVUS) was demonstrated to achieve better clinical outcomes of PCI [11] and is superior in evaluating the entire vessel measurement. However, in the treatment of calcified plaque, ultrasound waves are reflected by the surface of calcium; therefore, IVUS cannot provide quantitative evaluation of calcified plaque. Thus, this study aimed to evaluate the effect of crack formation pattern in calcified plaque after balloon angioplasty on final lumen area using OFDI.

## 2. Methods

### 2.1. Study Design and Patient Population

The Optical Frequency Domain Imaging-Guided Percutaneous Coronary Artery Intervention for Calcified Lesion registry is a physician-directed, noncompany sponsored, and retrospectively two-centered registry that enrolled consecutive patients who were undergoing PCI by using OFDI and DES for severely calcified coronary stenotic lesion. A severely calcified coronary stenotic lesion was defined angiographically according to the SYNTAX score. This score defines heavy calcification as multiple persisting opacifications, of the coronary wall, that are visible in more than one projection, surrounding the complete lumen of the coronary artery at the site of the lesion [12]. Fifty-eight patients who underwent PCI using OFDI for severely calcified coronary disease for the first time at two tertiary hospitals in Japan from January 2017 to June 2019 were enrolled. Eight patients who had no stents, no OFDI data before/after ballooning, or stenting, <180° of calcified plaque over the entire lesion length, or calcified nodule were excluded. Calcified nodule lesions were excluded according to their pathological difference from normal calcified plaque [13]. Calcified nodule was defined based on previous reports as “when fibrous cap disruption was detected over a calcified plaque characterized by protruding calcification, superficial calcium, and the presence of substantive calcium proximal and/or distal to the lesion” [14]. Thus, the study population consisted of 50 patients (54 lesions). The stent choice, use of debulking devices, type of balloons, poststenting dilatation, OFDI pullback speed, and dual antiplatelet therapy duration were decided upon the discretion of individual centers.

The research protocol was approved by the local ethics committee of all participating medical centers, which waived the requirement for obtaining informed consent from patients because of the retrospective nature of this study. We further excluded the patients who declined to participate in this study during the follow-up. All patient records were anonymized and deidentified before the analysis.

**Figure 1:**
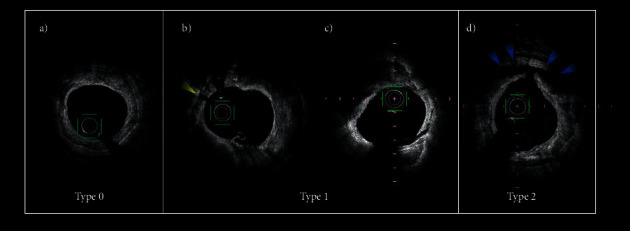
Crack formation pattern of calcified plaque. (a) Type 0, no cracks or injury in the vessel. (b, c) Type 1, with crack in calcified plaque (yellow arrowhead) or intima injury (red arrowhead) without medial dissection between calcified plaque and vessel wall. (d) Type 2, with crack in calcified plaque with medial dissection between calcified plaque and vessel wall (blue arrowhead).

In this study, we analyzed all OFDI data of patients who had PCI and compared the baseline characteristics and clinical outcomes among three types of crack formation in calcified plaque after balloon angioplasty: type 0, no crack in the plaque; type 1, no dissection between calcified plaque and vessel wall; and type 2, with any dissection between calcified plaque and vessel wall (Figure 1). OFDI can delineate media as a low-signal band. However, an assessment of media behind heavy calcium is difficult in some segments. Thus, it is hard to discriminate whether the dissection between calcium sheet and vessel wall is medial or adventitial. Hence, a type-2 crack was defined as any exfoliation of a calcium sheet from the vessel wall. OFDI measurements were performed using commercially available offline analysis software (LUNAWAVE, Terumo Corporation, Tokyo, Japan). The calcium component was detected according to previously validated criteria [15]. Each plaque was measured at approximately 3 mm intervals along a calcified lesion as long as the arc of the calcium was ≥180°. The lesion length was measured by OFDI. A total of 242 image slices were analyzed in this study. The same anatomic image slices were examined before and after balloon dilatation and after stenting. The timing of preballoon OFDI assessment in lesions treated with rotablator was just after rotational atherectomy with maximum burr size. The correspondence between OFDI imaging before, after balloon angioplasty, and after stenting (if postballooning was performed after stenting, then postballooning phase was analyzed) was identified by a synchronizing application that was a standard installation in LUNAWAVE. Regarding errors that might occur depending on the longitudinal movement of the OFDI catheter during the cardiac cycle, identification of corresponding frames in each phase was adjusted manually by the shape of calcified plaque or/and side branch location. In addition, crack of calcified plaque is easily affected longitudinally; therefore, we analyzed images approximately at 3 mm intervals. In case the correspondence of the frame was inaccurate, that frame was excluded from analysis.

All OFDI analyses were performed by two independent investigators, including a clinical engineering technologist and physician. The angle and thickness of calcified plaque and vessel morphology (lumen area and minor and major axis diameters of the lumen before balloon dilatation and after stenting) were quantitatively measured. Measuring the vessel area and diameter using OFDI is often challenging because of the thick calcified plaque; thus, vessel area was estimated from the immediate proximal and/or distal site at the image. The following quantitative measurements were obtained in preballooning phase: estimated vessel diameter and area, lumen major and minor axis diameter, lumen area, and calcium arc. In the postballooning phase, lumen major and minor axis diameter, lumen area, cracked calcium thickness, and crack patterns of calcified plaque were measured. In the poststenting phase, final lumen area and stent symmetry index (symmetry index = minimal stent diameter/maximal stent diameter) were also determined.

### 2.2. Definitions and Clinical Outcomes

We collected demographic, angiographic, and procedural data from hospital charts based on prespecified definitions. The primary outcome measure in this study was the area expansion ratio between preballooning and poststenting. The secondary outcome measures included symmetry index of implanted stents, thickness of cracked calcified plaque, and percentage of the final lumen area in the estimated vessel area. In the subanalysis, we compared the primary outcome measure according to calcium arc degrees (i.e., 180–224°, 225–269°, 270–314°, and 315–360°). Moreover, we analyzed the primary outcome measure only in the frame with the minimal lumen area (MLA) after procedure.

### 2.3. Statistical Analysis

Continuous variables are expressed as mean and standard deviation (SD), unless otherwise noted, and were compared using one-way analysis of variance or the Kruskal–Wallis test, depending on their distributions. Categorical variables are presented as numbers and percentages and were compared using the *χ*^2^ test. We evaluated the difference among the three types of crack formation in calcified plaque using the *t*-test. We investigated the affecting factors for area expansion ratio between preballooning and poststenting including multiple cracks, rotablator use, calcium arc, cutting balloon (CB) inflation pressure, balloon diameter/vessel diameter ratio, dissection beside calcium or crack inside it, and type of crack in calcified plaque based on prespecified definitions, using multivariate analysis of variance. To determine the optimal balloon size that could predict type-2 lesion modification, we evaluated the percentage of balloon diameter/mean vessel diameter among the lesions that could predict type-2 cracks among the full length of the lesion based on ROC curve analysis.

A receiver operating characteristic (ROC) curve analysis was performed. The cutoff point was defined as the greatest sum of sensitivity and specificity estimates.

Statistical analysis was conducted by a physician (HH) using the JMP 10.0 software (SAS Institute Inc., Cary, NC). All statistical analyses were two-tailed, and *P* < 0.05 was considered statistically significant. The authors had full access to and take full responsibility for the integrity of the data.

## 3. Results

### 3.1. Baseline Characteristics and Procedure Details

The study population reflected the real-world clinical practice, including large proportions of patients with advanced age, diabetes mellitus, multivessel disease, and high SYNTAX score (Table 1).

Regarding the lesion characteristics, majority of the lesions in this study were in the left anterior descending artery (LAD). The lesion length was 35.0 ± 15.9 mm. Initial passage of the OFDI imaging catheter (FastView^TM^, Terumo Corporation, Tokyo, Japan) through the lesion without rotational atherectomy or/and balloon angioplasty was successful in 56% of the cases, and rotational atherectomy was performed in 81% of the procedures. An orbital atherectomy system (OAS) was not used at all. All lesions were treated with a CB before stenting. The CB diameter was 2.74 ± 0.31 mm, and maximum pressure of CB dilatation was 10.4 ± 2.98 atm. All procedures were successful; no severe complications were observed (Table 2).

### 3.2. OFDI Analysis

Among 242 frame analysis, the discrepancy of crack type between 2 independent investigators was in 7 frames (*κ* = 0.95, 95% CI: 0.92–0.99). In these frames, inter- and intraobserver revalidated together and fixed data. In other analyzed parameters, inter- and intraobserver reproducibility was acceptable. Out of the 242 OFDI slices, 58 segments were type 0, 72 were type 1, and 112 were type 2. Estimated vessel area and diameter were significantly lower in type 2 (*p*=0.01). Lumen area before balloon dilatation was also significantly lower in type 2 (*p*=0.0003). The overall degrees of calcium arc were significantly higher in type 1 (*p*=0.0001). Thickness of cracked calcium was 465 ± 162 µm in type 1 and 387 ± 312 µm in type 2, and the maximum thickness was 820 µm. No significant differences in the symmetry index of the implanted stent (*p*=0.45) and the final lumen area (*p*=0.38) among the three groups were found (Table 3).

**Figure 2:**
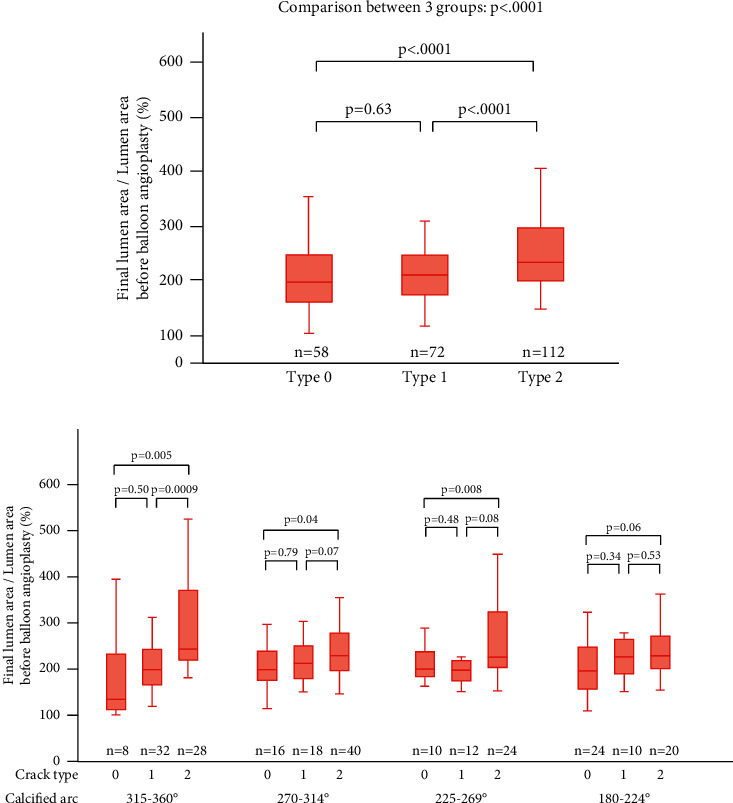
Lumen area expansion ratio between before ballooning and after stenting. (a) Lumen area expansion ratio between before ballooning and after stenting (all analyzed frames (*n* = 242)). Type 0, 196% (interquartile range (IQR), 163–244), type 1, 210% (IQR, 174–244), and type 2, 237% (IQR, 203–294). (b) Lumen area expansion ratio between before ballooning and after stenting according to the degrees of calcium arc. 315–360° calcium sections: type 0, 135% (IQR, 110–231); type 1, 197% (IQR, 167–241); and type 2, 242% (IQR, 220–368)). 270–314° calcium sections (type 0, 207% (IQR, 175–247); type 1, 214% (IQR, 177–255); and type 2, 227% (IQR, 200–280)). 225–269° calcium sections (type 0, 200% (IQR, 182–236); type 1, 199% (IQR, 175–220); and type 2, 232% (IQR, 212–334)). 180–224° calcium sections (type 0, 198% (IQR, 157–247); type 1, 228% (IQR, 189–265); and type 2, 231% (IQR, 201–269)).

The lumen area expansion ratio between preballooning and poststenting was significantly higher in type 2 than in other types (type 0, 196% (IQR, 163–244); type 1, 210% (IQR, 174–244); and type 2, 237% (IQR, 203–294) (Figure 2(a)). Regarding secondary outcome measure, percentage of the final lumen area in the estimated vessel area was significantly higher in type 2 than in other types (type 0, 51.2% (IQR, 43.1–58.4); type 1, 50.0% (IQR, 41.9–51.9); type 2, 56.9% (IQR, 47.0–64.3)) (Table 3, Supplementary Figure 1). In the subanalysis according to the degrees of calcium arc, the area expansion ratio between preballooning and poststenting was significantly higher in type 2 segments in 315–360° calcium sections (type 0, 135% (IQR, 110–231); type 1, 197% (IQR, 167–241); and type 2, 242% (IQR, 220–368)); 270–314° calcium sections (type 0, 207% (IQR, 175–247); type 1, 214% (IQR, 177–255); and type 2, 227% (IQR, 200–280)); and 225–269° calcium sections (type 0, 200% (IQR, 182–236); type 1, 199% (IQR, 175–220); and type 2, 232% (IQR, 212–334)). However, no significant difference among the three types was found in 180–224° calcium sections. (type 0, 198% (IQR, 157–247); type 1, 228% (IQR, 189–265); and type 2, 231% (IQR, 201–269) (Figure 2(b)). In the frame with MLA analysis, the lumen area expansion ratio between preballooning and poststenting was significantly higher in type 2 than in other types (type 0, 196% (IQR, 141–255); type 1, 210% (IQR, 186–245); and type 2, 243% (IQR, 207–295), that was the same trend with main analysis (Supplementary Figure 2).

**Figure 3:**
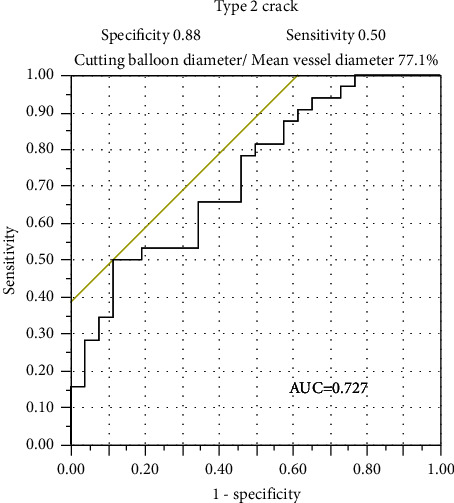
Receiver operating characteristic analysis for the prediction of type-2 cracks after balloon angioplasty. AUC, areas under the curve.

Multivariate analysis, which was performed to determine the factors that could significantly affect lesion modification, suggested degree of calcium arc, CB/vessel diameter ratio and type-2 crack had significantly influenced lumen area expansion after stenting compared with that before ballooning (Supplementary Table 1). Moreover, the CB size that could predict type-2 cracks among the full length of the lesion based on ROC curve analysis was 77.1% (CB diameter/mean vessel diameter among the lesion (sensitivity, 50.0%; specificity, 88.1%; area under the curve, 0.727; 95% confidence interval, −0.19–0.04; *p*=0.0009)) (Figure 3).

## 4. Discussion

The primary findings of this study are as follows: dissection between calcified plaque and the vessel wall is the most significant factor for lesion preparation of calcified coronary artery disease.

When performing PCI for severely calcified coronary disease, vessel preparation is essential for favorable short- and long-term clinical outcomes [16–18], and optical coherence tomography (OCT)/OFDI could be used to assess the mechanistic effects of atherectomy devices [19, 20]. A previous report suggested that lumen dilatation following balloon angioplasty was due not only to vessel wall dissection but also to the longitudinal displacement of the plaque [21]. However, calcified plaque is too hard to modify or compress by balloon dilatation. Therefore, to expand the area of the coronary artery with calcified plaque, the volume of calcified plaque should be reduced by using an atherectomy device or/and stretching the vessel arc without calcified plaque. In the present study, rotablator use was 81% (44 of 54 cases). The high usage rate was made by indication of OFDI for calcified lesion. In the participating 2 hospitals, operators tend to use OFDI for severely calcified lesion which seems to be needed debulking strategy from pre-PCI angiography. That made high frequency of rotational atherectomy strategy in the present study.

However elastic vessel arc without calcification could make elongation, excessive elongation may result in coronary perforation. A previous report demonstrated that rotational atherectomy before stenting is effective for calcified lesions [17, 18]; on the other hand, the effectiveness of arc elongation was not reported. We hypothesized that dissection between the calcified plaque and vessel wall is associated with a safe arc elongation and enlarged lumen area after stenting. Previous evidence for the effectiveness of CB for severely calcified lesions has been reported [22], and operators in the participating hospitals employed CB in all procedures. Type-2 group includes higher ratio of higher calcium arc degree. Operators in the participating hospitals might consider that using bigger size balloon may be safe without perforation in case of higher calcium arc degrees. However, few reports have evaluated the crack formation of calcified plaque using OCT/OFDI. A previous report, which included only calcified lesions treated with rotational atherectomy, suggested the best cutoff for the calcium arc and calcium thickness for the prediction of calcium crack were >227° and <670 *μ*m [16]. Although this study included both lesions treated with (81%) and without (19%) rotational atherectomy, as the calcium arc decreases, the ratio of no cracks increases. Furthermore, mean cracked calcium thickness was 465 µm in type 1 crack segments and 387 *μ*m in type-2 segments. These results have corresponded with a previous report.

The type-2 crack pattern of calcified plaque, i.e., dissection between calcified plaque and the vessel wall, was a significantly important factor for lesion preparation in both analysis of including all image frames and only in MLA frames, as hypothesized, particularly in segments with a high degree of calcified arc. Regarding the crack type may be in part depending on the morphology of calcified plaque, it was difficult to make definition of concentric/eccentric calcified plaque; however, PCI operators may feel the plaque as concentric that occupies larger degree of arc. Therefore, we performed subanalysis depending on calcium arc that was able to be evaluated quantitively. In the 315–360°group, type 2 crack was particularly significant for stent expansion, whereas 180–224°group was not. In the lower degree of calcium arc lesion, elastic vessel wall could extend well after stenting; therefore, the crack pattern may not be important in lower degree of calcium arc lesions.

**Figure 4:**
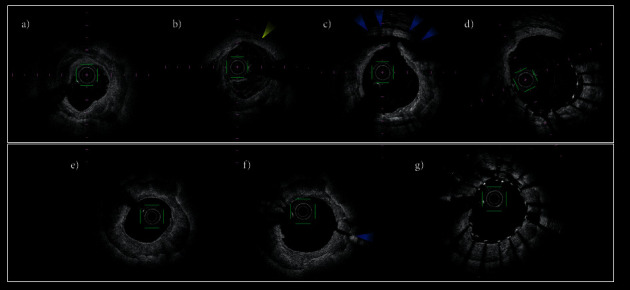
Representative cases of those who underwent optical frequency domain imaging (OFDI)-guided percutaneous coronary intervention (PCI) for severely calcified coronary disease. (a–d) A 69-year-old male with LAD lesion. Serial OFDI images obtained the same cross-section. (a) Initial OFDI image showed all rounded calcified plaque. (b) Postrotational atherectomy using a 1.5 mm and 2.0 mm burr; the calcium almost disappeared within the circumference (yellow arrowhead). The lumen area was 3.5 mm^2^ and the lumen perimeter 6.6 mm. (c) Postballooning using a cutting balloon (diameter, 3.0 mm; dilatation atmosphere, 10 atm; perimeter, 10.2 mm). Blue arrowheads show the medial dissection between calcified plaque and vessel wall. (d) Final OFDI image after stenting (DES diameter was 3.5 mm). The lumen area was 8.1 mm^2^, and the expansion ratio (final lumen area/lumen area before ballooning) was 231%. (e–g) A 74-year-old female with LAD lesion. Serial OFDI images obtained the same cross-section. Fast view catheter (TERUMO) did not pass the lesion. Thus, there was no initial OFDI image before rotational atherectomy. (e) Postrotational atherectomy using a 1.5 mm and 2.0 mm burr; the lumen was totally surrounded by thick calcium. The lumen area was 3.3 mm2 and the lumen perimeter 6.4 mm. (f) Postballooning using a cutting balloon (diameter, 2.25 mm; dilatation atmosphere, 8 atm; perimeter, 7.3 mm). The sheet calcium was cracked without medial dissection (blue arrowhead). (g) Final OFDI image after stenting (DES diameter was 2.5 mm). The lumen area was 4.6 mm^2^ and the expansion ratio (final lumen area/lumen area before ballooning) was 139%. DES, drug-eluting stent; LAD, left anterior descending artery; OFDI, optical frequency domain imaging; PCI, percutaneous coronary intervention.

Multivariate analysis showed CB/vessel diameter that might be effective in causing dissection was also a significantly important factor for lesion preparation. We showed that under 77.1% (cutting balloon diameter/mean vessel diameter) balloon size might not be favorable for vessel preparation in calcified lesion. Whereas, regarding there was no vessel perforation in this study population, we could not suggest the size limit of cutting balloon that could crack calcium significantly without vessel perforation (Figure 3). The low sensitivity for making type 2 crack might depend on the operators' discretion of not using too large size balloon to avoid vessel perforation. In this study, final lumen area was similar among the 3 groups. However, percentage of final lumen area in the estimated vessel area was significantly higher in type 2. Thus, type-2 crack may be a predictor for great lumen enlargement, whereas, in the whole length of stenosis lesion, type 2 crack might occur easier in smaller vessel segments than larger segments because balloon size/vessel diameter was relatively larger in smaller vessel segments. This might be a reason for the significant difference in estimated vessel area among 3 groups. Hence, in nearly all rounded calcified lesions, type-2 crack could result from balloon angioplasty with an optimal size balloon after reducing calcium volume using rotational atherectomy (Figures 4(a)–4(d)). Type-1 crack was not effective for lumen area expansion. Based on OFDI, calcium gaps resulting from balloon dilatation did not grow wider after stenting (Figures 4(e)–4(g)).

The final symmetry index of implanted stents was not different among three groups. Logically, high volume eccentric calcified lesion, that seems to have lower degree of calcified arc, has potential of low final stent symmetry index. On the other hand, lower degree of calcified arc location has rich elastic vessel wall which could elongate well after stenting. Hence, it is limited to discuss the result of symmetry index because of difficulty to quantitate calcified plaque volume and distribution in the vessel.

Whereas, final lumen area was not significantly different among the three groups; however, the ratio of final lumen area to estimated vessel area was significantly higher in type-2 crack group that was according to lower estimated vessel area in type-2 group. Therefore, these secondary outcome measures also suggest that type-2 crack was a feasible lesion preparation of calcified plaque.

While the ideal lesion preparation for severely calcified lesion was suggested in this study, in clinical practice, operators have difficulty in controlling the crack type of calcified plaque. Thus, optimal lesion preparation using atherectomy devices including rotational atherectomy, OAS, or/and special balloons must be explored. OFDI or OCT enables visualization of the crack pattern and has an important role in PCI for severely calcified coronary disease [23]. Furthermore, several previous reports discussed rotational atherectomy for the treatment of severe calcified coronary lesions. Routine lesion preparation by using rotational atherectomy did not reduce late lumen loss. However, rotational atherectomy was effective for initial procedural success particularly in complex lesion including tortuous artery, left main disease, and previous coronary artery bypass grafting [17]. Hence, the role of lithoplasty and OAS for calcified coronary lesion remains unclear [19, 24, 25].

This study has several limitations. First, this was a small number retrospective study. The procedure strategy had depended on operators, and thus, the final stent diameter or balloon diameter after stenting was not standardized with clear criteria, which in turn makes the actual maximum area expansion ratio between final lumen area and the lumen area before ballooning difficult to establish. Moreover, in this study, the stents used were not unified. The difference in radial force in each drug-eluting stent possibly affected the final lumen area in the calcified coronary artery. Second, majority of the lesions were in the LAD. Particularly, left circumflex artery lesion was only 3.7% of all the lesions. The left circumflex artery is often a tortuous vessel, and the indications for OFDI/OCT and atherectomy devices are limited. Hence, lesion preparation in other locations needs further investigation. Third, this study includes two lesion preparation groups, using both rotational atherectomy and balloon angioplasty group and angioplasty alone group. However, the aim of this study was to investigate the effectiveness of crack formation pattern of calcified plaque on stent expansion. Therefore, we included these two groups in the same analysis. Forth, there are no follow-up data of enrolled patients in this study. Fifth, this study did not include quantitative coronary angiography (QCA) data analysis. Therefore, further investigation including whether successful lesion preparation corresponds to target lesion revascularization or/and adverse cardiac events is required. Fifth, we suggested cutting balloon size to make optimal crack for calcified lesion. However, it was difficult to compare 4-blade and 3-blade cutting balloon because only one lesion which was used 4-blade cutting balloon.

Despite these limitations and limited availability of OFDI in the world, from a clinical perspective, evaluation of lesion modification for calcified plaque using optical intracoronary imaging has possibility to improve clinical outcome of severely calcified coronary lesion.

## 5. Conclusion

In conclusion, dissection between calcified plaque and the vessel wall is a significant factor for a satisfactory final lumen area after stenting.

## Figures and Tables

**Table 1:** Baseline clinical characteristics.

Variable	*n* = 50
Age (years)	74.9 ± 7.9
Male	34 (68%)
BMI (kg/m^2^)	23.4 ± 3.8
Hypertension	46 (92%)
Dyslipidemia	36 (72%)
Current smoker	9 (18%)
Diabetes mellitus	27 (54%)
Atrial fibrillation	4 (8%)
LVEF (%)	63.8 ± 12.2
ESRD on HD	3 (6%)
eGFR≤60 without HD (mL/min/1.73 m^2^)	25 (50%)
PAD	6 (12%)
COPD	1 (2%)
Aortic disease	1 (2%)
Prior PCI	21 (42%)
Prior CABG	2 (4%)
Prior stroke	6 (12%)
CHF	7 (14%)
Prior MI	18 (36%)
SYNTAX score	21.5 ± 22.9
SYNTAX2 score (PCI)	34.3 ± 15.9
Acute coronary syndrome	5 (10%)
Multivessel disease	27 (54%)

Medications	
Aspirin	49 (98%)
Thienopyridine	50 (100%)
OAC	5 (10%)
Statins	43 (86%)
*β*-blockers	25 (50%)
ACE-I/ARB	30 (60%)

Data are presented as number (%) or mean ± standard deviation, unless otherwise noted. ACE-I, angiotensin-converting-enzyme inhibitor; ARB, angiotensinI receptor blocker; BMI, body mass index; CABG, coronary artery bypass grafting; CHF, congestive heart failure; COPD, chronic obstructive pulmonary disease; eGFR, estimated glomerular filtration rate; ESRD, end-stage renal disease; HD, hemodialysis; LVEF, left ventricular ejection fraction; MI, myocardial infarction; OAC, oral anticoagulants; PAD, peripheral artery disease; PCI, percutaneous coronary intervention.

**Table 2:** Baseline lesion characteristics and procedure details.

Variable	*n* = 54
Target vessel	
Isolated LMCA	0
LMCA + LAD	2 (3.7%)
LAD	39 (72%)
LCx	2 (3.7%)
RCA	11 (20%)
Lesion length (mm)	35.0 ± 15.9
Tortuous vessel	12 (22%)
CTO	0
Rotational atherectomy	44 (81%)
CB diameter (mm)	2.74 ± 0.31
Maximum pressure of CB dilatation (atm)	10.4 ± 2.98
Rotablator burr size (mm) ^†^	1.78 ± 0.20
Slow flow/no reflow during procedure	5 (9.3%)

Data are presented as number (%) or mean ± standard deviation, unless otherwise noted. CTO, chronic total occlusion; LAD, left anterior descending artery; LCx, left circumflex artery; LMCA, left main coronary artery; RCA, right coronary artery; CB, cutting balloon. ^†^Calculated in 44 lesions.

**Table 3:** OFDI analysis.

Variable	Type 0 (*n* = 58)	Type 1 (*n* = 72)	Type 2 (*n* = 112)	*p* value
Estimated vessel diameter (mm)	4.05 ± 0.49	4.09 ± 0.51	3.89 ± 0.46	0.01
Estimated vessel area (mm^2^)	13.04 ± 3.26	13.27 ± 3.17	12.03 ± 2.74	0.01
Major axis diameter before using CB (mm)	2.36 ± 0.37	2.29 ± 0.37	2.11 ± 0.46	0.0004
Minor axis diameter before using CB (mm)	1.79 ± 0.31	1.72 ± 0.31	1.57 ± 0.31	0.0001
Lumen area before using CB (mm^2^)	3.37 ± 1.02	3.10 ± 0.91	2.73 ± 1.01	0.0003
Calcium arc (°)	251 ± 56	294 ± 62	277 ± 53	0.0001
Calcium arc 315–360°	8 (14%)	32 (44%)	28 (25%)	0.0009
Calcium arc 270–314°	16 (28%)	18 (25%)	40 (36%)	0.25
Calcium arc 225–269°	10 (17%)	12 (17%)	24 (21%)	0.67
Calcium arc 180–224°	24 (41%)	10 (15%)	20 (18%)	0.0005
Major axis diameter after using CB (mm)	2.56 ± 0.32	2.63 ± 0.42	2.67 ± 0.41	0.22
Minor axis diameter after using CB (mm)	1.99 ± 0.28	1.99 ± 0.28	1.95 ± 0.29	0.52
Lumen area after using CB (mm^2^)	4.08 ± 0.87	4.21 ± 1.08	4.17 ± 1.15	0.80
Cracked calcium thickness (µm)	N/A	465 ± 162 (*n* = 42)	387 ± 312 (*n* = 26)	0.08
Final lumen area (mm^2^)	6.43 ± 1.31	6.38 ± 1.36	6.66 ± 1.51	0.38
Symmetry index	0.86 ± 0.09	0.84 ± 0.08	0.86 ± 0.08	0.45
Final lumen area/estimated vessel area (%)	51.2 ± 12.4	50.0 ± 12.2	56.9 ± 13.0	0.0005

Data are presented as number (%) or mean ± standard deviation, unless otherwise noted. CB, cutting balloon; OFDI, optical frequency domain imaging.

## Data Availability

The data used to support the findings of this study are available from the corresponding author upon request.

## Supplementary Materials

Supplementary Materials Supplementary Figure 1: final lumen area/estimated vessel area (%). Type 0, 50.0% (interquartile range (IQR), 43.4–58.7), type 1, 48.6% (IQR, 41.8–54.4), and type 2, 53.9% (IQR, 47.0–64.3). Supplementary Figure 2: lumen area expansion ratio between before ballooning and after stenting (MLA frames only). Type 0, 196% (interquartile range (IQR), 141–255), type 1, 210% (IQR, 187–245), and type 2, 243% (IQR, 207–295). Supplementary Table 1: multivariate analysis of lesion modification factors. The affecting factors for area expansion ratio between preballooning and poststenting.
