# Supplementary material for: Effect of Crack Patterns in Calcified Plaque on Lumen Area after Stenting for a Severe Calcified Coronary Artery (from the Optical Frequency Domain Imaging-Guided Percutaneous Coronary Artery Intervention for Calcified Lesion Registry)
Source: J Interv Cardiol. 2022 Feb 27;2022:7821956. doi: 10.1155/2022/7821956 (PMC8898875; doi:10.1155/2022/7821956)
Supplement: Supplementary Materials — Supplementary Figure 1: final lumen area/estimated vessel area (%). Type 0, 50.0% (interquartile range (IQR), 43.4–58.7), type 1, 48.6% (IQR, 41.8–54.4), and type 2, 53.9% (IQR, 47.0–64.3). Supplementary Figure 2: lumen area expansion ratio between before ballooning and after stenting (MLA frames only). Type 0, 196% (interquartile range (IQR), 141–255), type 1, 210% (IQR, 187–245), and type 2, 243% (IQR, 207–295). Supplementary Table 1: multivariate analysis of lesion modification factors. The affecting factors for area expansion ratio between preballooning and poststenting. [file 7821956.f1.zip › 7821956.f1/Supplementary Table 1.docx]

Supplementary Table 1

| Variable | P value |
| --- | --- |
| Multiple cracks | 0.66 |
| Calcium arc | 0.004 |
| Rotational atherectomy before balloon angioplasty | 0.06 |
| Inflation pressure of cutting balloon | 0.73 |
| Balloon diameter/ Vessel diameter | <0.0001 |
| Dissection beside calcium or crack inside calcium | 0.28 |
| Type 2 crack | 0.002 |
